# Cortical Activation during Landmark-Centered vs. Gaze-Centered Memory of Saccade Targets in the Human: An FMRI Study

**DOI:** 10.3389/fnsys.2017.00044

**Published:** 2017-06-23

**Authors:** Ying Chen, J. D. Crawford

**Affiliations:** ^1^Center for Vision Research, York University, TorontoON, Canada; ^2^Departments of Psychology, Biology, and Kinesiology and Health Science, York University, TorontoON, Canada; ^3^Canadian Action and Perception Network, TorontoON, Canada; ^4^Vision: Science to Applications Program, York University, TorontoON, Canada

**Keywords:** landmark-centered coding, directional selectivity, gaze-centered coding, fMRI, saccade

## Abstract

A remembered saccade target could be encoded in egocentric coordinates such as gaze-centered, or relative to some external allocentric landmark that is independent of the target or gaze (landmark-centered). In comparison to egocentric mechanisms, very little is known about such a landmark-centered representation. Here, we used an event-related fMRI design to identify brain areas supporting these two types of spatial coding (i.e., landmark-centered vs. gaze-centered) for target memory during the *Delay phase* where only target location, not saccade direction, was specified. The paradigm included three tasks with identical display of visual stimuli but different auditory instructions: *Landmark Saccade* (remember target location relative to a visual landmark, independent of gaze), *Control Saccade* (remember original target location relative to gaze fixation, independent of the landmark), and a non-spatial control, *Color Report* (report target color). During the *Delay phase*, the *Control* and *Landmark Saccade* tasks activated overlapping areas in posterior parietal cortex (PPC) and frontal cortex as compared to the color control, but with higher activation in PPC for target coding in the *Control Saccade* task and higher activation in temporal and occipital cortex for target coding in *Landmark Saccade* task. Gaze-centered directional selectivity was observed in superior occipital gyrus and inferior occipital gyrus, whereas landmark-centered directional selectivity was observed in precuneus and midposterior intraparietal sulcus. During the *Response phase* after saccade direction was specified, the parietofrontal network in the left hemisphere showed higher activation for rightward than leftward saccades. Our results suggest that cortical activation for coding saccade target direction relative to a visual landmark differs from gaze-centered directional selectivity for target memory, from the mechanisms for other types of allocentric tasks, and from the directionally selective mechanisms for saccade planning and execution.

## Introduction

To explore and interact with the visual world, people make frequent saccades toward both visible and remembered targets (Enright, 1995; Rayner, 1998, 2009; Henderson and Hollingworth, 1999; Henderson et al., 1999; Land et al., 1999; Land and Hayhoe, 2001; Henderson, 2003; Bays and Husain, 2007). In the absence of additional cues, visual movement targets can be encoded in memory with respect to egocentric frames of reference, such as gaze-, head- or body-centered (Schlag and Schlag-Rey, 1987; Schall, 1991; Dassonville et al., 1995; Andersen, 1997; Karn et al., 1997; Colby, 1998; Henriques et al., 1998; Tehovnik et al., 1998, 2000; Burnod et al., 1999; Klier et al., 2001; Cohen and Andersen, 2002; Martinez-Trujillo et al., 2004; Monteon et al., 2013). However, the addition of other stable visual stimuli, independent of gaze, provides potential cues for an allocentric (world-centered) frame of reference for coding target location (Carrozzo et al., 2002; Obhi and Goodale, 2005; Crawford et al., 2011; Tatler and Land, 2011; Sharika et al., 2014). These cues can be implicit, such as the influence of general background information on a memory-guided movement (Mohrmann-Lendla and Fleischer, 1991; Whitney et al., 2003; Uchimura and Kitazawa, 2013), or they can be explicit, such as the deliberate choice of remembering target location relative to another cue that is judged to be stable (Olson, 2003; Krigolson and Heath, 2004; Krigolson et al., 2007; Cordova et al., 2012). In the case where the allocentric cue is independent of the goal as opposed to part of the same object (as well as any egocentric frame) it is often called a ‘landmark’ (Diedrichsen et al., 2004; Obhi and Goodale, 2005; Krigolson et al., 2007; Byrne et al., 2010). Psychophysical studies in the reach system suggest that when both egocentric and allocentric cues are present, human subjects use an optimal combination of both, weighted through a combination of reliability and subjective judgments of landmark stability (Byrne and Crawford, 2010). However, such behavioral and computational studies cannot reveal the functional neuroanatomy of these systems. The goal of the current study was to compare human cortical activation for gaze-centered coding of remembered saccade targets vs. the explicit coding of a saccade target relative to a specified visual landmark, independent of gaze (landmark-centered).

The neural correlates of egocentric mechanisms for saccades are relatively well known, based on findings from both neurophysiological and human imaging studies. It has been shown that posterior parietal cortex (PPC), frontal eye field (FEF), and supplementary eye field (SEF) are involved in the coding of remembered saccadic targets and planning in egocentric reference frames (Colby, 1998; Sereno et al., 2001; Andersen and Buneo, 2002; Munoz, 2002; Medendorp et al., 2003, 2005a,b; Munoz and Everling, 2004; Schluppeck et al., 2005; Curtis and D’Esposito, 2006; Kastner et al., 2007; Van Pelt et al., 2010; Crawford et al., 2011; Kravitz et al., 2011). Although we did not test between egocentric reference frames in the current study, most previous fMRI studies found a contralateral gaze-centered topography (target direction relative to gaze) in human midposterior intraparietal sulcus (mIPS) and FEF (Sereno et al., 2001; Medendorp et al., 2003, 2005a,b; Schluppeck et al., 2005; Curtis and D’Esposito, 2006; Kastner et al., 2007; Van Pelt et al., 2010). However, these studies did not compare cortical activation for egocentric coding to allocentric coding.

Neuroimaging studies of allocentric coding have mainly focused on visual and cognitive mechanisms of spatial judgment (Fink et al., 1997, 2000; Honda et al., 1998; Galati et al., 2000; Committeri et al., 2004; Neggers et al., 2006; McKyton and Zohary, 2007; Zaehle et al., 2007), although two more recent studies involved aiming movements of the hand (Thaler and Goodale, 2011; Chen et al., 2014). The results of most of these studies are generally consistent with the neuropsychology-based view that the ventral visual stream is necessary for the allocentric coding of target locations, as opposed to the dorsal stream egocentric mechanisms described above (Goodale and Milner, 1992; Schenk, 2006). However, one fMRI study identified a region in parietal cortex involved in an ‘automatic’ allocentric coding of visual stimuli relative to large background stimuli (Uchimura et al., 2015), and another found allocentric vs. egocentric timing differences in premotor cortex responses (Postle et al., 2003).

Relatively little is known about the cortical mechanisms specific for saccade planning and target coding using an independent allocentric landmark. Several neurophysiological studies for object-centered (target location relative to a part of the object itself) spatial coding of saccade targets revealed selective activity in SEF (Olson and Gettner, 1995, 1996; Olson and Tremblay, 2000; Olson, 2003). However, to our knowledge, no previous neuroimaging study has compared the allocentric and egocentric mechanisms for saccade target coding, and no study of any kind has investigated cortical activity for target location relative to an independent visual landmark, in terms of either general cortical activation patterns or the directional selectivity of specific areas.

Based on the previous literature from reach and cognitive tasks, one might expect that egocentric and allocentric mechanisms for saccade target memory might have both shared and distinct cortical mechanisms (Galati et al., 2000; Zaehle et al., 2007; Thaler and Goodale, 2011; Chen et al., 2014). More specifically, one might expect the involvement of occipital cortex for the gaze-centered directional selectivity of saccade target memory as shown in our recent fMRI study for reach (Chen et al., 2014). If the cortical areas involved in allocentric directional selectivity of saccade targets are similar to those in object-centered coordinates, one might expect higher activation in SEF, or other areas in frontal cortex as indicated in previous neurophysiological studies (Olson and Gettner, 1996; Olson and Tremblay, 2000). If the cortical regions involved in the allocentric directional selectivity for the coding of saccade targets are similar to those for reach targets, one might expect an engagement of temporal cortex (Chen et al., 2014). However, if there are specific cortical areas involved in the allocentric directional selectivity for saccade targets relative to independent visual landmarks, i.e., effector-specific mechanisms, one might expect the activation of areas that differ from those involved in either the coding of landmark-centered reach targets or object-centered saccade targets.

To test these predictions, we used an event-related fMRI paradigm similar to our recent reach study (Chen et al., 2014), which consisted of two saccade tasks and one non-spatial color control task (1) to examine brain areas involved in spatial coding of remembered saccade targets in landmark-centered, (gaze-independent) and gaze-centered (landmark-independent) frames of reference; (2) to investigate which brain areas show directional selectivity of remembered saccade targets in these two coordinates; (3) to compare gaze-centered directional selectivity for remembered saccade targets vs. actual saccades during the motor response. In our experimental design all visual stimuli displayed in the three tasks were identical, but the instruction for each task was different so that any observed differences on cortical activity would be based on the instruction. Our results showed that cortical areas for the coding of remembered saccade targets in gaze-centered coordinates were different from those employed for coding in landmark-centered coordinates, in both general activation and direction specificity. The cortical areas showing gaze-centered directional selectivity during the delay phase differed from those during the response phase. The cortical areas showing landmark-centered directional selectivity of saccade target memory were different from those observed for reach targets and saccade targets represented in object-centered coordinates (Olson and Gettner, 1995; Olson and Tremblay, 2000; Chen et al., 2014), suggesting an effector- and coordinate-dependent mechanisms for allocentric coding of target direction.

## Materials and Methods

### Participants

Twelve right-handed participants (nine females and three males, aged 22–42 years) participated in this study and gave informed consent prior to the experiment. All had normal or corrected to normal vision and had no known neuromuscular deficits. We chose this set of subjects based on their general experimental experience, level of motivation, and precedents for subject numbers set in similar studies of visuomotor control in healthy subjects (Cavina-Pratesi et al., 2007; Gallivan et al., 2011). The resulting dataset was sufficient to yield statistically significant results that survived corrections for multiple comparisons, tested our hypotheses, and generally agreed with expectations based on the literature (see Results). This study was approved by the York Human Participants Review Subcommittee.

### Experimental Apparatus and Stimuli

We used a same apparatus as that in a previous reach study (Chen et al., 2014). The visual stimuli of light dots produced by optic fibers were embedded in a custom-built board mounted atop a platform. The platform was placed above the abdomen of the participant and affixed to the scanner bed. The board was approximately perpendicular to the direction of gaze on the central fixation point and was placed about ∼60 cm away from the eyes of the participants. Participant’s head was slightly tilted to allow direct viewing of the stimuli without using mirrors. An eye-tracking system (iView X) was used in conjunction with the MRI-compatible Avotec Silent Vision system (RE-5701) to record gaze position from the right eye during fMRI experiments. A button pad was placed on the left side of the participant’s abdomen and used as a response key for the *Color Report* task (see Experimental Paradigm and Timing). Participants wore headphones to hear auditory instructions about the upcoming trial. During the experiment, participants were surrounded by complete darkness, except for the visual stimuli, which were illuminated from behind the screen by optic fibers.

**FIGURE 1:**
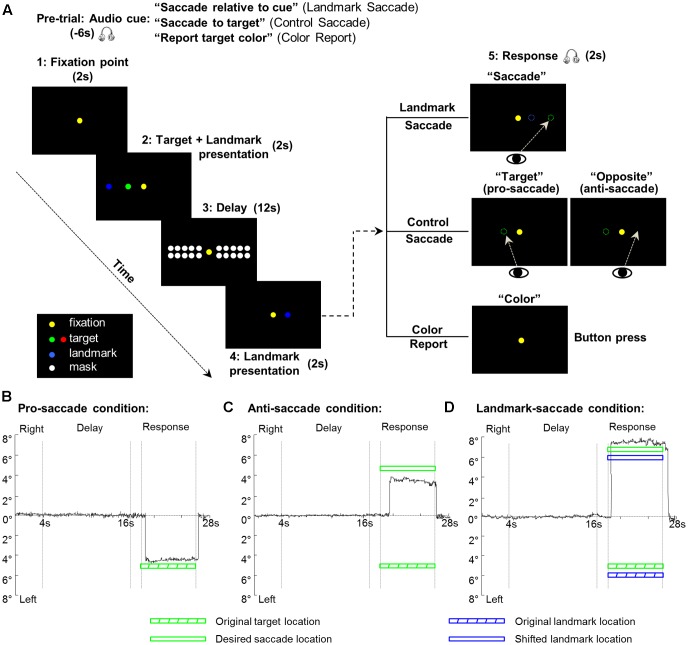
Experimental paradigm and eye trajectories for each of the three saccade conditions from one representative participant. **(A)** The display of the visual stimuli is identical for the three tasks (Landmark Saccade, Control Saccade, and Color Report). The critical difference between the two saccade tasks is the reference frames used for the coding of target location for the upcoming saccade. In the Landmark Saccade task, target location is encoded relative to the landmark. In the Control Saccade task, target location is encoded relative to initial gaze position. In the Color Report task, the color of the target, rather than location, is being remembered and reported. **(B)** Eye trajectory for the pro-saccade condition in the Control Saccade task. The first and second vertical gray lines indicate onset and offset of the delay phase, respectively. The third and fourth vertical gray lines indicate start of a saccade response and end of the saccade by making gaze back to the central fixation point. **(C)** Eye trajectory for the anti-saccade condition in the Control Saccade task. Legends as in **(B)**. **(D)** Eye trajectory for the Landmark Saccade task. Legends as in **(B)**.

There were four types of stimuli each presented in a different color: yellow for the central fixation point, green or red for the saccade targets, blue for the visual landmarks, and white for the mask (**Figure 1A**). The dots of light corresponding to targets and relative visual landmarks were located to the left and the right of the central fixation point with a visual angle of four to seven degrees on each side, and being separated from each other by one visual degree. These dots could be red, green or blue, therefore they could be used as a target or a visual landmark in different trials. This allowed us to create 40 different combinations of target and visual landmark locations where the target could be located one or two visual degrees to the left or to the right of a visual landmark. Initial target and visual landmark were both displayed either to the left or to the right of the central fixation point. Since the central fixation point was always fixed at midline and aligned with participants’ initial gaze/head/body position, we used the initial gaze as the zero point for analysis of gaze-centered directional selectivity (i.e., target/saccade right of gaze = target/saccade right of midline, target/saccade left of gaze = target/saccade left of midline).

The mask consisted of 20 dots of light displayed in two rows, one above and one below the targets. The location of each dot for the mask was aligned with the midpoint between two adjacent targets dots. The purpose of using a mask was to avoid potential after effects arising from the illumination of the target and the landmark in the dark. Since our analyses focused mainly on the *Delay phase*, it was critical to ensure that the recruitment of target location for the upcoming saccade was resulting from memory rather than utilizing the afterimage of the target.

### Experimental Paradigm and Timing

We employed an event-related design to investigate three main questions. First, we contrasted the brain regions involved in the general processing of saccade target location in landmark-centered vs. gaze-centered frames of reference during the *Delay phase*. Second, we determined the brain areas showing directional selectivity of spatial coding of saccade targets in gaze-centered vs. landmark-centered coordinates during the same *Delay phase*. Third, we investigated the areas involved in processing saccade direction during the *Response phase*.

The paradigm consisted of three tasks: *Control Saccade* (*CS*), *Landmark Saccade* (*LS*), and *Color Report* (*CR*) (**Figure 1A**). Note that the visual stimuli (spatial locations, colors, and timing) used in these three tasks were identical; only the auditory instructions differed. In the *Landmark Saccade* task, participants had to remember the location of the target relative to a visual landmark that was independent of gaze (and the fixation light), or any other egocentric cue. The saccade target and the additional landmark were initially presented together, and then the landmark re-appeared at the same or at a different location, in the same or opposite visual hemifield. After that, participants made a saccade to the remembered target location relative to the re-presented landmark (see **Figure 1A** and below for details). In the *Control Saccade* task, participants had to remember the original target location relative to gaze, and then make a saccade to the remembered target, either at its initial location (*pro-saccade*), or at its mirror location in the opposite hemi-field (*anti-saccade*). In the *Control Saccade task* the landmark location was irrelevant to solve the task and the fixation point was redundant with gaze direction. The *anti-saccade* condition was used to equalize the motor aspects in the *CS* and *LS* tasks: in both tasks, subjects could remember stimulus direction during the *Delay Phase*, but the probability of the final saccade being in the same or different hemifield was equal and completely unpredictable, so subjects could not yet plan the correct saccade direction. Other types of directionally non-specific motor preparation, including random or bi-directional saccade plans, should be revealed in our general activity analysis, but subtracted out in the directional analyses described below. The *Color Report* task was used as a non-spatial control where participants only reported the color of targets by pressing a button once or twice corresponding to the green or red saccade target. Eye trajectories from one trial under each of the three saccade conditions (CS: pro-saccade, CS: anti-saccade, LS) from one representative participant are plotted through time in **Figures 1B–D**, respectively.

Our paradigm consisted of five phases (*Fixation point, Target and Landmark presentation, Delay, Landmark presentation, Response*) (**Figure 1A**). Prior to each trial, a recorded auditory instruction signaled the participant about the upcoming task: “Saccade relative to cue” (for *Landmark Saccade* tasks), “Saccade to target” (for *Control Saccade* tasks), “Report target color” (for *Color Report* tasks). Therefore, participants always knew what task they were going to perform for the upcoming trial. Each trial started with the presentation of the central fixation for participants to fixate throughout the experiment. After 2 s, a target was presented along with a landmark for 2 s. Depending on the initial instruction, after the target and landmark disappeared, during the following 12 s *Delay phase* participants had to remember the location of the target relative to the landmark (*Landmark Saccade*), the location of the target regardless of the landmark (*Control Saccade*), or the color of the target (*Color Report*). After the delay, the landmark re-appeared for 2 s either at its original location or at a novel location in the same or opposite hemifield of its first presentation. Subsequently, an auditory signal cued participants to saccade toward the target location in the landmark-centered coordinates (audio: “Saccade”), i.e., the target location relative to the re-presented landmark location for the *Landmark Saccade* task. In the *Control Saccade* task, participants were instructed to saccade toward the target location in the gaze-centered coordinates (audio: “Target” for *pro-saccade*), or the location opposite to the egocentric target location (audio: “Opposite” for *anti-saccade*). In the *Color Report* task, participants indicated the color of the previously presented target by pressing the button (audio: “Color”). Each run contained 18 trials where each task was repeated six times in a random order. An intertrial interval of 16 s was added between each trial to allow the hemodynamic response to return to baseline, thus a run time was approximately 12 min in length. Each participant was tested in six runs including 36 trials for each task. Participants were trained to perform the tasks 1 day prior to scan.

In addition to the three tasks, we considered directional selectivity of remembered saccade targets in the horizontal dimension in gaze-centered (i.e., Left vs. Right relative to gaze fixation) and landmark-centered coordinates (i.e., Left vs. Right relative to the visual landmark) during the delay phase. This gave rise to three factors: 3 Tasks (*CS, LS, CR*) × 2 Target locations relative to initial gaze (*Left of Gaze: LG, Right of Gaze: RG*) × 2 Target locations relative to landmark (*Left of Landmark: LL, Right of Landmark: RL*). Therefore, there were 12 combinations (conditions) during the delay phase: *CS: LG:LL, CS: LG:RL, CS: RG:LL, CS: RG:RL, LS: LG:LL, LS: LG:RL, LS: RG:LL, LS: RG:RL, CR: LG:LL, CR: LG:RL, CR: RG:LL, CR: RG:RL*. Among those, there were six conditions for gaze-centered or landmark-centered directional selectivity (i.e., gaze-centered: *LG or RG* × *CS*, *LS*, or *CR*; landmark-centered: *LL* or *RL* × *CS*, *LS*, or *CR*) so that we recorded 18 trials for each condition.

Note that our experiment does not discriminate between different types of egocentric coding (i.e., subjects could use gaze-, head-, or body- centered cues in the Control Saccade task), but most other studies which have discriminated between these favored gaze-centered codes for saccades (Sereno et al., 2001; Medendorp et al., 2003, 2006; Van Pelt et al., 2010), so unless otherwise stated, we assume this gaze-centered view as a default theory of egocentric codes for saccades. Note also that we maintained the fixation light on at all times in every task. This was done both to enforce fixation, and so that visual and motor activity related to fixation would be equal and subtract out across tasks in all of the voxelwise subtractions described below. We considered the possibility that this stimulus might provide an additional allocentric cue in both of our experimental tasks. While this is possible, it is not a confound in our experimental design because: (1) in the Control Saccade task, the fixation point always aligned with gaze (again, likely the dominant reference frame for saccades) as well as head and body midline in our set-up. Thus, in this task the fixation target at best provides redundant information that would always be present in natural gaze behaviors. (2) In the Landmark Saccade task (gaze-independent), the fixation point is irrelevant for solving the task, because the subject is required to remember the location of the target relative to a landmark that will randomly, and unpredictably appear in either the left or right visual hemifield, independent of the fixation target. Ultimately, we were able to segregate cortical activation in these two coordinates (gaze-centered vs. landmark-centered) using this method (see Results).

### Behavioral Analysis

Following our fMRI experiments, we inspected eye position data for every trial to ensure that participants correctly followed all instructions. Errors in eye movements were defined as trials in which participants made a saccade toward the target or the visual landmark, or were not able to maintain central fixation during the delay phase, or the location of the saccade endpoint was on the opposite side of the actual target location relative to the midline on the touch screen. Trials that showed those errors were modeled as confound predictors and excluded from further fMRI analyses (see Data Analyses). All participants completed at least 96 correct trials (89% of the total trials).

In order to confirm that participants actually used egocentric or additional landmark visual information in the corresponding task (*CS* or *LS*) to encode target location as instructed, and to exclude the possibility that they simply made saccades to the correct side of the screen midline, we performed a correlation analysis. First, we calculated the signed distance between a participant’s saccade response for a given trial and the screen midline, then calculated the signed distance between the proper target location (whether defined in gaze-centered or landmark-centered coordinates) and the screen midline. If participants made saccades toward the correct location, these two values should be well correlated in both *CS* and *LS* tasks. The across-subject means of these correlation coefficients were 0.85 ± 0.01 for the *CS* task and 0.87 ± 0.01 for the *LS* task. We then applied Fisher’s r-to-z transformation to the individual subject correlation coefficients (r) so that we could use standard *t*-tests to compare the between-subjects means of *z*-values to zero. If participants were using the gaze-centered or landmark-centered spatial information for target coding, then these coefficients should have been significantly greater than zero. Standard *t*-tests showed that mean of correlation coefficient was significantly greater than zero in both tasks (*p*_cs_ = 0.0000001, *p*_ls_ = 0.0000001). The correlations were still significant (*p*_cs_ = 0.000003, *p*_ls_ = 0.000004) when absolute values for the distance were used, showing that subjects also adjusted the amplitude of the saccades in response to different target amplitudes on each side.

### Imaging Parameters

This study was conducted at the neuroimaging center at York University using a 3-T whole body MRI system (Siemens Magnetom TIM Trio, Erlangen, Germany). The posterior half of a 12-channel head coil (6 channels) was placed at the back of the head in conjunction with a 4-channel flex coil covering the anterior part of the head. The former was tilted at an angle of 20° to allow the direct viewing of the stimuli.

Functional data were acquired using an EPI (echo-planar imaging) sequence (repetition time [TR] = 2000 ms; echo time [TE] = 30 ms; flip angle [FA] = 90°; field of view [FOV] = 192 mm × 192 mm, matrix size = 64 × 64 leading to in-slice resolution of 3 mm × 3 mm; slice thickness = 3.5 mm, no gap; 35 transverse slices angled at approximately 25° covering the whole brain). The slices were collected in ascending and interleaved order. During each experimental session, a T1-weighted anatomical reference volume was acquired using a MPRAGE sequence (TR = 1900 ms; TE = 2.52 ms; inversion time TI = 900ms; FA = 9°; FOV = 256 mm × 256 mm × 192 mm, voxel size = 1 mm × 1 mm × 1 mm).

### Preprocessing

Data were analyzed using the Brain Voyager QX 2.2 software (Brain Innovation, Maastricht, The Netherlands). The first two volumes of each fMRI scan were discarded to avoid T1 saturation effects. For each functional run, slice scan time correction (cubic spline), temporal filtering (removing frequencies < 2 cycles/run) and 3D motion correction (trilinear/sinc) were performed. The 3D motion correction was performed aligning each volume to the volume of the functional scan closest to the anatomical scan. Following inspection of the 3D motion correction parameters, the runs showing abrupt head motion exceeding 1 mm or 1° were discarded. Two data sets from two runs, one for each participant, were discarded from the analyses due to head motion exceeding our set threshold. The functional run closest to the anatomical image for each participant was co-registered to the anatomical image. Functional data were then mapped into standard Talairach space, using the spatial transformation parameters from each participant’s anatomical image. Subsequently, functional data was spatially smoothed using a FWHM of 8 mm.

### fMRI Analyses

For each participant, we used a general linear model (GLM) including 26 predictors in total. In particular, we used one predictor for the *Target and Landmark presentation phase* (2 s or 1 volume). We used 12 predictors (12 s or 6 volumes), one for each condition of target directional selectivity in the *Delay phase*, (see Experimental Paradigm and Timing). Moreover, we added four predictors for gaze-centered directional selectivity of saccades during the delay phase: 2 Saccade tasks (CS, *LS*) × 2 Saccade direction relative to gaze (*Left of Gaze: LG, Right of Gaze: RG*) to confirm a lack of saccade directional specificity in the delay phase. We used one predictor (2 s or 1 volume) for the *Landmark presentation phase.* There were two factors in the *Response phase* (2 s or 1 volume): 3 Tasks (*CS, LS, CR*) × 2 Saccade direction relative to gaze (*LG, RG*). This resulted in six predictors: *Control Saccade: LG, Control Saccade: RG, Landmark Saccade: LG, Landmark Saccade: RG, Color Report: LG, Color Report: RG*, thus allowing us to explore the brain areas involved in processing the saccade direction during response. We used one predictor for keeping eyes on the saccade target (6 s or 3 volumes) for the current response to ensure stable saccade performance, and one predictor for shifting gaze back to the central fixation point for the next trial (2 s or 1 volume). Each predictor was derived from a rectangular wave function convolved with a standard hemodynamic response function (HRF), the Brain Voyager QX’s default double-gamma HRF. In addition, we added six motion correction parameters and errors made in eye data as confound predictors.

We performed contrasts on beta weights (β) using a group random effects (RFX) GLM where percentage signal change transformation had been performed. Our study aimed to explore brain areas encoding the saccade target location during the *Delay phase* prior to the movement. First, we used Contrast no. 1: [(Delay CS + Delay LS) > Delay CR] to investigate areas involved in coding of target location for the *Control Saccade* and *Landmark Saccade* tasks as compared to the *Color Report* control task. We collapsed the target location left and right to gaze and landmark in the *Delay phase*. Second, we performed Contrast no. 2: [Delay LS > Delay CS] to identify brain areas involved in processing target location in landmark-centered vs. gaze-centered coordinates during the *Delay phase.* Third, we performed Contrast no. 3: [Delay CS (Target Right of Gaze) > Delay CS (Target Left of Gaze)] to examine areas showing directional selectivity of target location relative to gaze. We collapsed left and right target locations relative to landmark. Fourth, we performed Contrast no. 4: [Delay LS (Target Right of Landmark) > Delay LS (Target Left of Landmark)] to investigate brain areas showing directional selectivity of target location relative to landmark. In this contrast, we collapsed left and right target locations relative to gaze. Finally, we tested whether areas in the parieto-frontal saccade network show a preference for saccades made to the targets in the contralateral visual hemifield during the *Response phase*, not during *the Delay phase.* This was assessed by Contrast no. 5: [Saccade Right of Gaze > Saccade Left of Gaze] in *Control Saccade* and *Landmark Saccade* tasks, respectively, during the *Response* and *the Delay phases*. For this contrast, direction was defined as the saccade direction relative to gaze.

**FIGURE 2:**
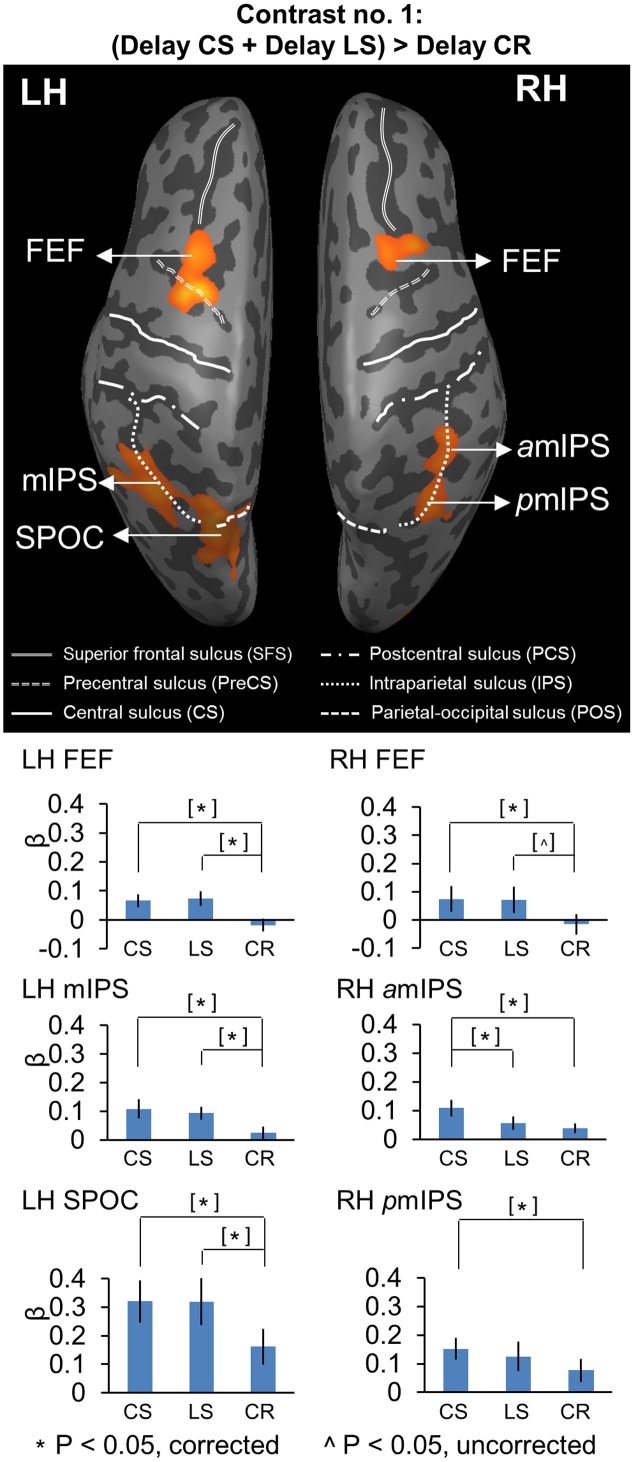
Voxelwise statistical map obtained with the RFX GLM and activation levels for each area using Contrast no. 1 [(Delay CS + Delay LS) > Delay CR]. Top panel: activation map rendered on the inflated brain of one representative participant. Bottom panel: bar graphs show the β weights for the three tasks in each area. CS, Control Saccade task. LS, Landmark Saccade task. CR, Color Report task. [^∗^] Significant difference between two tasks for *p* < 0.05, non-independent of the criteria used to select the area. [ˆ] Significant difference between two tasks for *p* < 0.05, uncorrected, non-independent of the criteria used to select the area. Error bars indicate 95% confidence intervals. FEF corresponds to the anatomic area at the intersection between precentral sulcus and superior frontal sulcus, and was classified based on the neuroimaging literature on saccades (Paus, 1996; Luna et al., 1998).

Activation maps for group voxelwise results were rendered either on the inflated anatomical image of one representative participant (**Figures 2**, **9**) or on the average anatomical MRI from twelve participants (**Figures 4**, **5**, **7**, **8**). In order to correct for multiple comparisons, we performed a cluster threshold correction (Forman et al., 1995) using BrainVoyager’s cluster-level statistical threshold estimator plug-in. This algorithm uses Monte Carlo simulations (1000 iterations) to estimate the probability of a number of contiguous voxels being active purely due to chance while taking into consideration the average smoothness of the statistical maps. Areas that did not survive a cluster threshold correction were excluded from further analyses. The estimated minimum cluster size was 28 voxels (3 mm^3^) for a total volume of 756 mm^3^. Subsequently, a Bonferroni correction was applied to paired-sample *t*-tests on β weights extracted from each area that survived the cluster threshold correction. The Bonferroni correction was performed for three comparisons (corrected *p* = 0.0167) aimed at answering our main questions.

**FIGURE 3:**
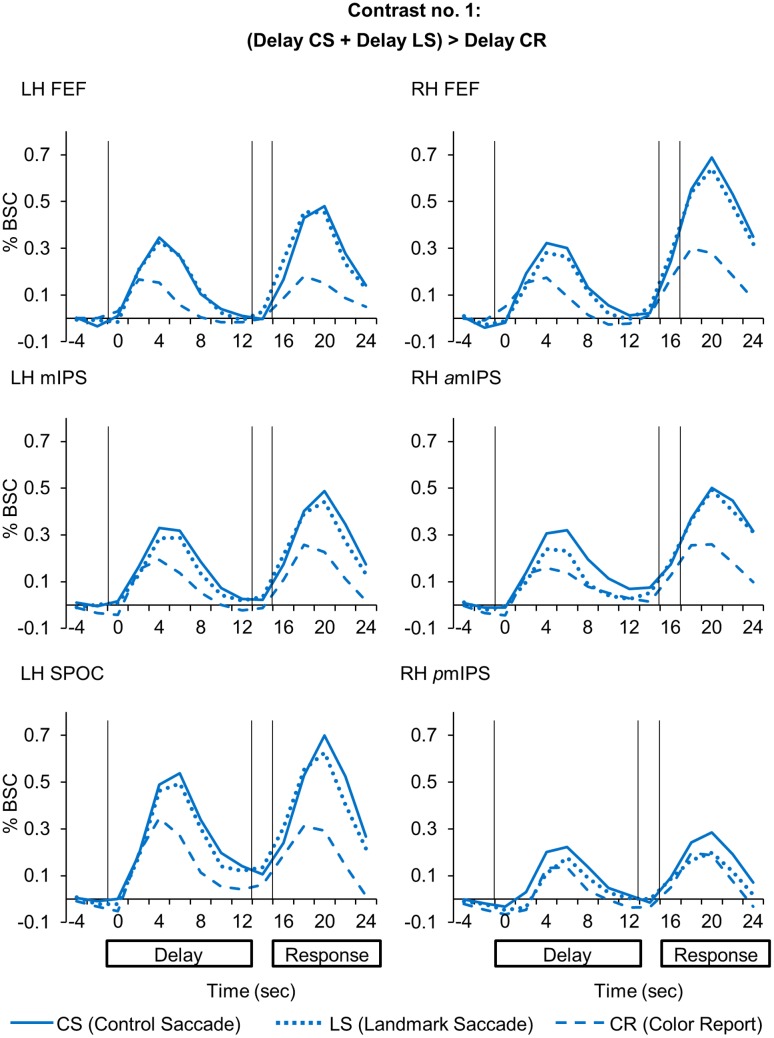
Time course data in line graphs show average % BSC from each of regions identified using Contrast no. 1 [(Delay CS + Delay LS) > Delay CR].

**FIGURE 4:**
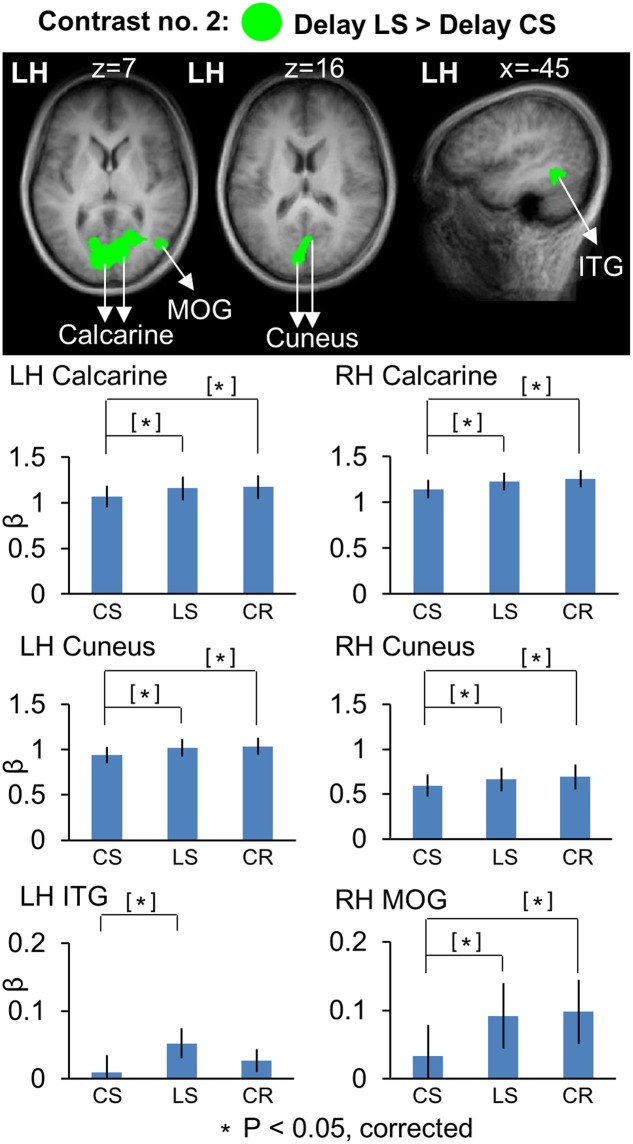
Voxelwise statistical map obtained with the RFX GLM and activation levels for each area using Contrast no. 2 [Delay LS > Delay CS]. Top panel: activation map overlaid on the averaged anatomical image from all participants. Bottom panel: bar graphs show the β weights for the three tasks in each area. Legends as in **Figure 2**.

**FIGURE 5:**
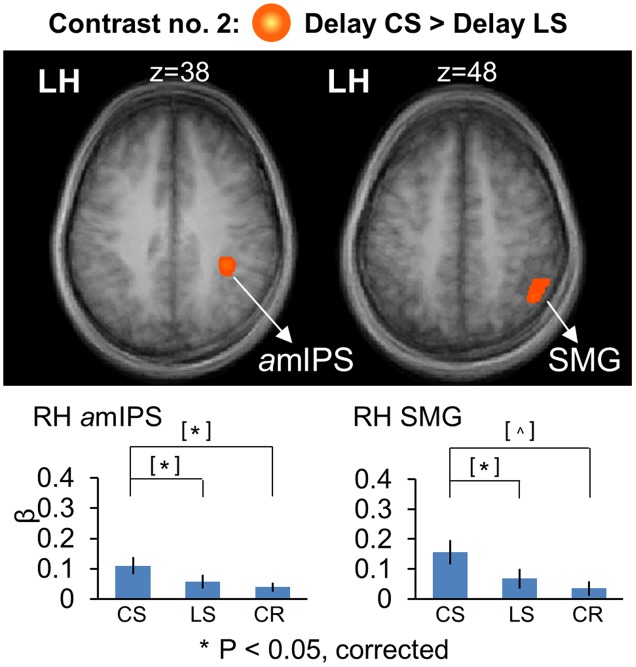
Voxelwise statistical map obtained with the RFX GLM and activation levels for each area using Contrast no. 2 [Delay CS > Delay LS]. Top panel: activation map overlaid on the averaged anatomical image from all participants. Bottom panel: bar graphs show the β weights for the three tasks in each area. Legends as in **Figure 2**.

**FIGURE 6:**
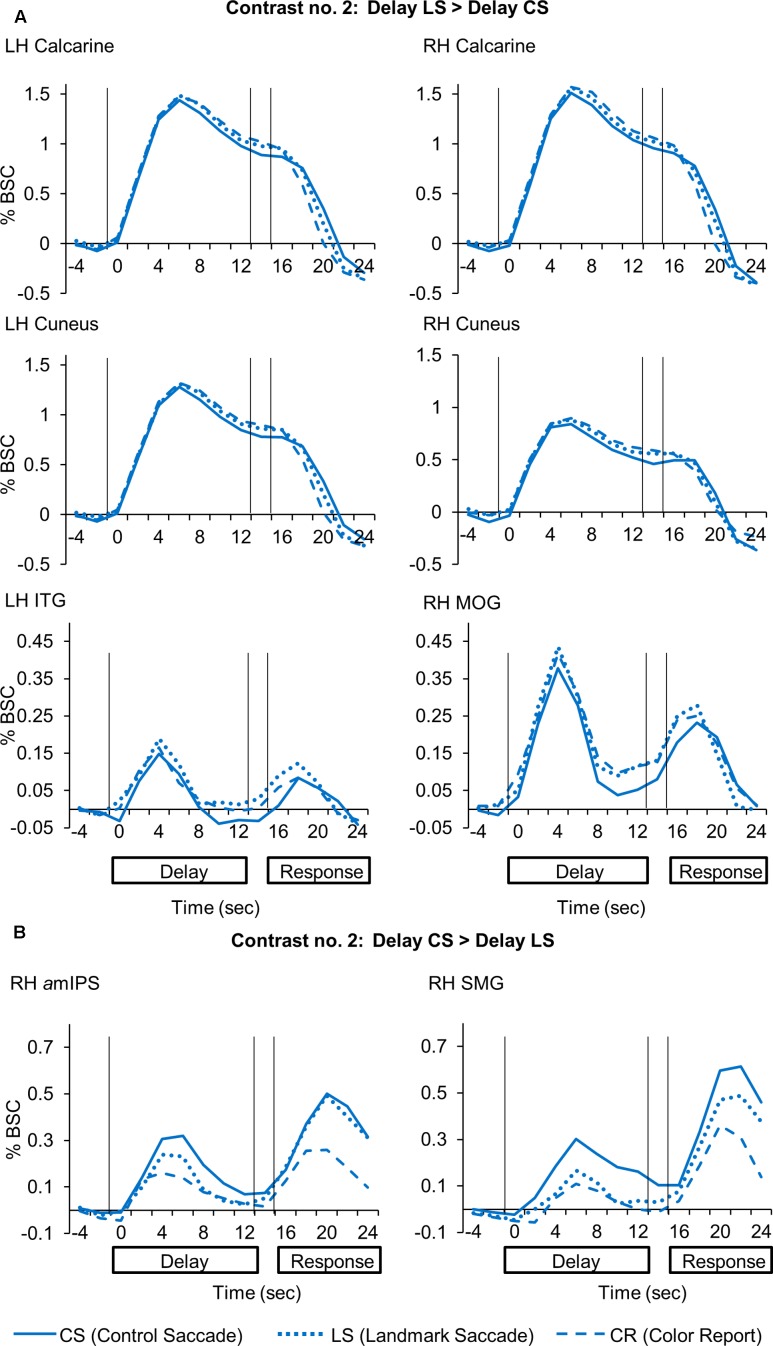
Time course data in line graphs show average % BSC from each of regions identified using Contrast no. 2. **(A)** [Delay LS > Delay CS]. **(B)** [Delay CS > Delay LS].

**FIGURE 7:**
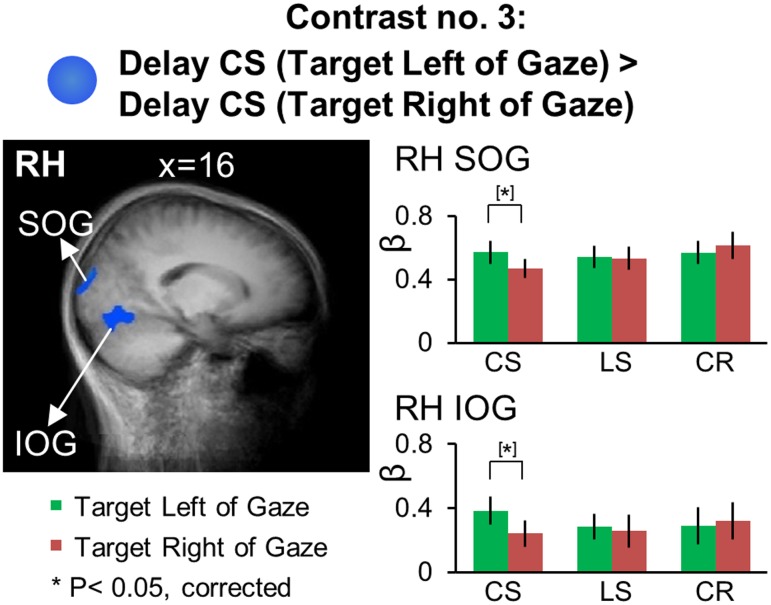
Voxelwise statistical map obtained with the RFX GLM and activation levels for each area using Contrast no. 3, [Delay CS: (Target Left of Gaze) > Delay CS: (Target Right of Gaze)]. Left panel: activation map overlaid on the averaged anatomical image from all participants. Right panel: bar graphs show the β weights for each condition in each area. Legends as in **Figure 2**.

For Contrasts no. 1 and 2, we performed the following comparisons on β weights: *CS* vs. *CR*, *LS* vs. *CR, CS* vs. *LS* to explore the difference of brain activity between tasks. For Contrasts no. 3, we performed three comparisons on β weights: *RG* vs. *LG* in *CS*, *LS*, and *CR* tasks, respectively, to investigate whether the coding of left and right target location relative to gaze was specific to the *Control Saccade* task or it also existed in other two tasks. For Contrasts no. 4, we performed three comparisons on β weights: *RL* vs. *LL* in *CS*, *LS*, and *CR* tasks, respectively, to examine whether the coding of target location relative to the landmark was specific to the *Landmark Saccade* task or it also applied to other two tasks. For Contrasts no. 5, we performed three comparisons on β weights: Saccade *RG* vs. Saccade *LG* in *CS*, *LS*, and *CR* tasks, respectively, to confirm that the coding of saccade direction relative to gaze only emerged in the two saccade tasks, not in the *Color Report* control task during the *Response phase*. The results on β weights are plotted in bar graphs in **Figures 2**, **4**, **5**, **7**, **9** to illustrate significant differences between conditions at the corrected *p*-value, unless specified (see Results). Results that are non-independent of the selection criteria are indicated in square brackets in the β weight plots. The time course from each brain region identified using contrast nos. 2 and 3 were plotted in **Figures 3**, **6** to show the percentage of BOLD signal change (% BSC) over the delay and response periods among the three tasks.

## Results

The key question behind our design was to compare cortical activity involved in the coding of saccade targets in gaze-centered and landmark-centered coordinates during the *Delay phase.* As shown in **Figure 1A**, in this phase only target direction was specified (in gaze-centered or landmark-centered frames of reference), whereas saccade direction was informed only at the end of *Landmark presentation phase* through the re-presented landmark (*Landmark Saccade)*, or the *pro/anti-saccade* instruction (*Control Saccade*). We performed a detailed analysis for the *Delay phase*, followed by a brief analysis on saccade directional coding during the *Response phase.* The result from each contrast is shown in figures with a voxelwise analysis of whole brain activity first, and then followed by further paired *t*-tests on β weights and/or time courses between conditions from each significant activity cluster. See **Table 1** for a list of the cortical areas that were active in these analyses, and their acronyms.

**Table 1:** Acronyms for brain areas identified by voxelwise analyses.

Acronyms	Names of brain areas
*a*IOG	Anterior inferior occipital gyrus
*a*mIPS	Anterior midposterior intraparietal sulcus
FEF	Frontal eye field
IOG	Inferior occipital gyrus
ITG	Inferior temporal gyrus
LOtG	Lateral occipitotemporal gyrus
MFG	Middle frontal gyrus
mIPS	Midposterior intraparietal sulcus
MOG	Middle occipital gyrus
MTG	Middle temporal gyrus
*p*IOG	Posterior inferior occipital gyrus
*p*IPS	Posterior intraparietal sulcus
*p*mIPs	Posterior midposterior intraparietal sulcus
SEF	Supplementary eye field
SMG	Supramarginal gyrus
SOG	Superior occipital gyrus
SPL	Superior parietal lobule
SPOC	Superior parieto-occipital cortex


### Task-Related Cortical Activation during the Delay Phase

We performed Contrast no. 1 [(Delay CS + Delay LS) > Delay CR] to explore the brain areas showing higher activation in the two experimental saccade tasks (*CS, LS*), as opposed to the non-spatial control task (*CR*). **Figure 2** shows the resulting activation map, superimposed on an inflated cortical surface, with the corresponding mean β weights for each task and area plotted beneath as bar graphs. The Talairach coordinates of these brain areas are reported in **Table 2**. Note that the activations revealed by this contrast might be related to any aspect of target coding, including landmark location coding, and/or general motor preparation with expectancy in an upcoming saccade, except target or movement direction (this is dealt with in subsequent sections).

**Table 2:** Talairach coordinates and number of voxels for contrast no. 1.

	Talairach coordinates			
		
Brain areas	*x*	*y*	*z*	No. of voxels
**[(Delay CS + Delay LS) > Delay CR]**			
LH FEF	-24	-1	50	513
RH FEF	28	3	50	508
LH mIPS	-40	-48	42	512
RH *a*mIPS	33	-37	39	353
RH *p*mIPS	33	-57	39	392
LH SPOC	-18	-67	49	511


Compared to the *Color Report* task, the *Control* and *Landmark Saccade* tasks elicited higher activation in: bilateral FEF, mIPS and superior parieto-occipital cortex (SPOC) in the left hemisphere, anterior (*a*mIPS) and posterior mIPS (*p*mIPS) in the right hemisphere (**Figure 2**, upper panel). Paired *t*-tests on β weights (**Figure 2**, lower panels) indicated higher *CS* vs. *CR* activation in these areas: bilateral FEF [LH: *t*_(11)_ = 5.33, *p* = 0.00024; RH: *t*_(11)_ = 3.56, *p* = 0.0045], left mIPS [*t*_(11)_ = 2.95, *p* = 0.013], left SPOC [*t*_(11)_ = 3.30, *p* = 0.0071], right *a*mIPS [*t*_(11)_ = 3.27, *p* = 0.0075] and right *p*mIPS [*t*_(11)_ = 3.01, *p* = 0.012]. We also found higher activation for *LS* vs. *CR* in some of these areas: bilateral FEF [LH: *t*_(11)_ = 4.66, *p* = 0.00070; RH: *t*_(11)_ = 2.76, *p* = 0.018], left mIPS [*t*_(11)_ = 4.17, *p* = 0.0016] and left SPOC [*t*_(11)_ = 3.24, *p* = 0.0079]. In addition, the *t*-tests also indicated higher activation for *CS* vs. *LS* in right *a*mIPS [*t*_(11)_ = 2.88, *p* = 0.015]. In summary, this analysis mostly revealed overlapping activation in the *Control* and *Landmark Saccad*e tasks in bilateral FEF, left mIPS and left SPOC, except that right *a*mIPS showed higher activation for *CS* vs. *LS* tasks, and that right *p*mIPS showed higher activation for *CS* vs. *CR* tasks. In **Figure 3**, the time course of each region shows a consistent pattern of BOLD signal change over the delay phase, indicating preparatory activity for saccades (CS, LS) diverges from passive visual activation (CR) at 2 s of the delay period.

Subsequently, we used Contrast no. 2 to directly compare *LS* and *CS* activation during the *Delay phase* to explore the areas showing higher activation for *LS* vs. *CS* tasks and vice versa. The Talairach coordinates for these brain areas are reported in **Table 3**. The areas showing significantly higher activation in the *LS* task are showed on both horizontal and sagittal brain slices (**Figure 4**). This analysis identified several areas in occipital cortex showing higher activation for the *LS* vs. *CS* tasks, including bilateral calcarine sulcus [LH: *t*_(11)_ = 3.64, *p* = 0.0039; RH: *t*_(11)_ = 3.47, *p* = 0.0053] and cuneus [LH: *t*_(11)_ = 3.74, *p* = 0.0032; RH: *t*_(11)_ = 3.02, *p* = 0.012], and middle occipital gyrus (MOG) in the right hemisphere [*t*_(11)_ = 3.44, *p* = 0.0055]. However, our *t*-test analysis on β-weights (lower panels) showed that these occipital areas also showed higher activation for *CR* vs. *CS*, including bilateral calcarine sulcus [LH: *t*_(11)_ = 2.96, *p* = 0.013; RH: *t*_(11)_ = 3.08, *p* = 0.010] and cuneus [LH: *t*_(11)_ = 3.01, *p* = 0.012; RH: *t*_(11)_ = 3.55, *p* = 0.0046], and right MOG [*t*_(11)_ = 3.15, *p* = 0.0093]. This suggests that general activation of early visual cortex was not specific to any one of the tasks in the current experiment. In addition, this analysis (**Figure 4**) revealed higher activation in left inferior temporal gyrus (ITG) [*t*_(11)_ = 3.13, *p* = 0.0096] in the *LS* than the *CS* task. This area also showed higher activation in the *LS* than the *CR* task, but this did not reach significance [*t*_(11)_ = 1.61, *p* = 0.14].

**Table 3:** Talairach coordinates and number of voxels for contrast no. 2.

	Talairach coordinates			
		
Brain areas	*x*	*y*	*z*	No. of voxels
**Delay LS > Delay CS**			
LH ITG	-45	-48	-8	400
LH Calcarine	-4	-82	5	512
RH Calcarine	5	-78	5	510
LH Cuneus	-2	-84	10	730
RH Cuneus	4	-67	10	323
RH MOG	46	-68	8	410
**Delay CS > Delay LS**			
RH *a*mIPS	33	-37	39	353
RH SMG	43	-49	48	431


**Figure 5** shows the areas that showed significantly higher activation in the *CS* task, using similar conventions to **Figure 4**, but overlaid on horizontal brain slices and with the corresponding β-weights for each area and task plotted beneath. These areas included right *a*mIPS [*t*_(11)_ = 2.88, *p* = 0.015], and right supramarginal gyrus (SMG) [*t*_(11)_ = 2.90, *p* = 0.014]. The comparison of β-weights for these regions showed that they were also significantly activated more than the *Color Report* task [*CS* vs. *CR*: right *a*mIPS: *t*_(11)_ = 3.27, *p* = 0.0075, right SMG: *t*_(11)_ = 2.43, *p* = 0.033]. This demonstrates that activation of amIPS and SMG showed specificity for the *Control Saccade* task. The time course from each of the occipital and temporal regions in **Figure 6A** shows that the instruction-dependent difference of activation starts as early as 4 s during the delay phase, and the time course from each of the two posterior parietal regions shows such an activation divergence even earlier, at 2 s of the delay period (**Figure 6B**).

To summarize, these results showed overlapping parieto-frontal areas for spatial memory of saccade target representation in gaze-centered and landmark-centered reference frames. In addition, the *Control Saccade task* evoked higher activation in parieto-frontal areas such as *a*mIPS and SMG. In contrast, the *Landmark Saccade task* produced higher activation in temporal cortex (ITG) and early visual cortex areas such as calcarine and cuneus, although the latter areas also showed activity in the *Color Report* task.

### Gaze-Centered Directional Selectivity: Target Location Relative to Gaze during the Delay Phase

We used Contrast no. 3 [Delay CS (Target Right of Gaze) > Delay CS (Target Left of Gaze)] to investigate areas showing gaze-centered directional selectivity during the *Delay phase.* The Talairach coordinates of these brain areas are reported in **Table 4**. We did a two-tailed contrast, but we found no active voxels showing higher activation for right vs. left target location. However, as illustrated in **Figure 7**, areas superior occipital gyrus (SOG) [*t*_(11)_ = 4.21, *p* = 0.0015] and inferior occipital gyrus (IOG) [*t*_(11)_ = 3.92, *p* = 0.0024] in the right hemisphere showed higher activation for targets to the left vs. right of gaze. Analysis of the β-weights for SOG and IOG (**Figure 7**, right column) showed no gaze-centered directional selectivity in either the *LS* or *CR* tasks. To confirm that the gaze-centered directional selectivity described above was specific to the *Control Saccade* task throughout the brain, we performed a full-brain voxelwise contrast [Delay LS (Target Right of Gaze) > Delay LS (Target Left of Gaze)] during the *Delay phase* in the *LS* task (not shown). There were no significantly active voxels for this contrast.

**Table 4:** Talairach coordinates and number of voxels for contrast no. 3 and no. 4.

	Talairach coordinates			
		
Brain areas	*x*	*y*	*z*	No. of voxels
**Delay CS (Target Left of Gaze) > Delay CS (Target Right of Gaze)**			
RH SOG	16	-97	7	236
RH IOG	16	-79	-13	467
**Delay LS (Target Right of Landmark) > Delay LS (Target Left of Landmark)**			
LH precuneus	-14	-68	36	247
RH precuneus	10	-63	36	230
LH mIPS	-40	-36	36	472
**Delay LS (Target Left of Landmark) > Delay LS (Target Right of Landmark)**			
RH calcarine	7	-78	8	360


In summary, we found significant gaze-centered directional selectivity in SOG and IOG during the *Control Saccade* task, suggesting that these early visual areas are specifically involved in the coding of remembered saccade target location in gaze-centered reference frames.

### Allocentric Directional Selectivity: Target Location Relative to Landmark during the Delay Phase

The key point of this study was that our design allowed us to investigate cortical regions for the coding of saccade targets relative to a visual landmark in the *Landmark Saccade* task.

**FIGURE 8:**
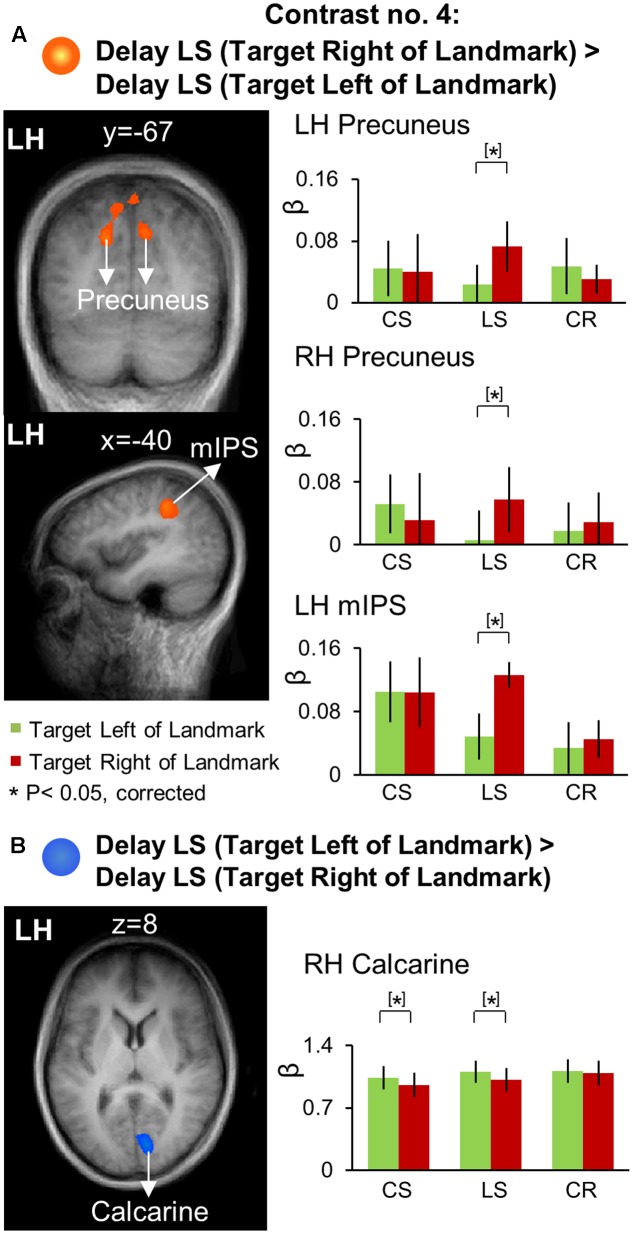
Voxelwise activation maps obtained with the RFX GLM and activation levels for each area using Contrast no. 4. **(A)** [Delay LS (Target Right of Landmark) > Delay LS (Target Left of Landmark)]. **(B)** [Delay LS (Target Left of Landmark) > Delay LS (Target Right of Landmark)]. Left panels: activation maps overlaid on the averaged anatomical image from all participants. Right panels: bar graphs show the β weights for each condition in each area. Legends as in **Figure 2**.

We used Contrasts no. 4 [Delay LS (Target Right of Landmark) > Delay LS (Target Left of Landmark)] to identify brain areas involved in landmark-centered directional selectivity. Talairach coordinates of these brain areas are reported in **Table 4**. The results of this analysis are shown in **Figure 8**, with activation clusters superimposed on anatomical brain slices in the left column and the results of further *t*-test analysis of β weights on the right. This figure separates areas that show rightward (A) and leftward (B) tuning with respect to the landmark.

As shown in **Figure 8A**, this contrast revealed significantly higher rightward activation in bilateral precuneus [LH: *t*_(11)_ = 4.33, *p* = 0.0012; RH: *t*_(11)_ = 3.88, *p* = 0.0026] and left mIPS [*t*_(11)_ = 3.66, *p* = 0.0037]. Our β weight comparisons (right column) revealed that in parietal cortex this directional selectivity was specific to the *LS* task. This analysis also revealed higher leftward activation in the right calcarine sulcus (**Figure 8B**) for the *LS* task [*t*_(11)_ = 3.28, *p* = 0.0074] as well as for the *CS* task [*t*_(11)_ = 2.95, *p* = 0.013].

In order to examine the brain regions showing landmark-centered directional selectivity only in the *Landmark Saccade* task (i.e., task-specificity), we performed voxelwise contrast [(Target Right of Landmark) > (Target Left of Landmark)] in the *Delay phase* for the *Control Saccade* task. This confirmed left vs. right directional selectivity in right calcarine sulcus, and revealed additional occipital areas (not shown) including bilateral IOG and left calcarine sulcus, but those areas were not task specific.

In summary, precuneus and mIPS showed significant landmark-centered directional selectivity only for the *Landmark Saccade* task, suggesting that dorsal-medial PPC and middle IPS are specifically recruited for the coding of remembered saccade targets in the landmark-centered reference frame (see Discussion).

### Saccade Direction Coding during the Response Phase

Although the main purpose of this study was to examine landmark-centered vs. gaze-centered directional coding of saccade targets during the *Delay Phase*, for comparing to the previous egocentric saccade literature and testing the effect of recalculating saccade vector using a shifted landmark on motor activation, we also examined saccade direction coding during the *Response Phase.* As noted above, we did not observe gaze-centered directional selectivity in the parietal-frontal saccade circuit during the *Delay phase*. The reason may be that unlike previous fMRI studies where saccade direction could be planned during memory delay (Medendorp et al., 2005b; Kastner et al., 2007), participants in our study would not be able to plan the horizontal position of the actual saccade in the *Delay phase* until saccade direction was specified by either the reappearance of the landmark (*LS* task) or the *pro/anti* instruction (*CS* task) right before the *Response phase*. To confirm this, we performed Contrast no. 5 [Saccade Right of Gaze > Saccade Left of Gaze] in *Control* and *Landmark Saccade* tasks, respectively, during the *Delay* and *Response phases*.

**FIGURE 9:**
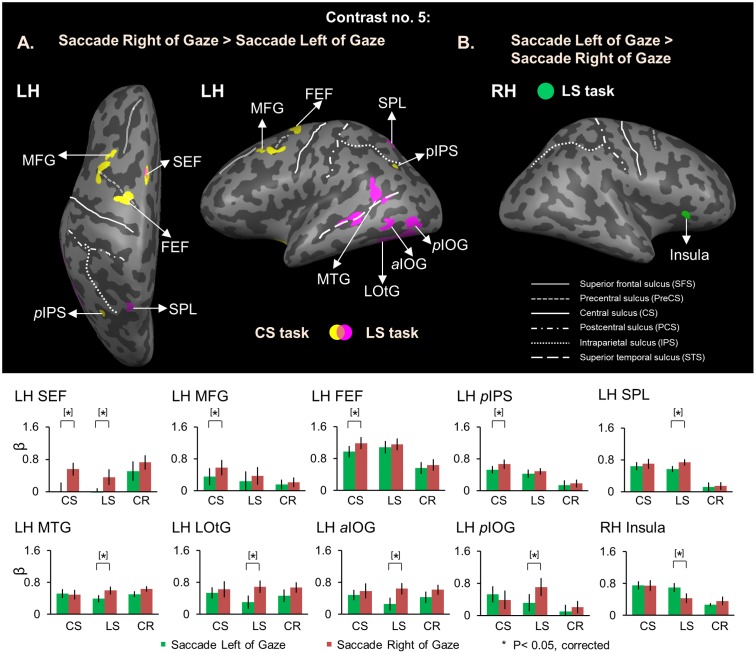
Voxelwise activation maps obtained with RFX GLM and activation levels for each area using Contrast no. 5 during the Response phase. Top panel: activation maps rendered on the inflated brain of one representative participant. **(A)** Saccade Right of Gaze > Saccade Left of Gaze. Yellow, voxels activated in the CS task. Pink, voxels activated in the LS task. Orange, voxels activated in both tasks. **(B)** Saccade Left of Gaze > Saccade Right of Gaze. Green, voxels activated in the LS task. Bottom panel: bar graphs show the β weights for each condition in each area. Legends as in **Figure 2**. FEF and SEF correspond to anatomic areas, the intersection between precentral sulcus and superior frontal sulcus (FEF), and the medial surface of the superior frontal gyrus (SEF), respectively. Note that to be consistent with previous saccade neuroimaging literature, we have provided functional rather than anatomic names for these structures, based on previously published coordinates (Luna et al., 1998; Cornelissen et al., 2002; Connolly et al., 2007).

As expected, no voxels showed significant activation for this saccade direction selectivity during the *Delay Phase*, whereas analysis of the *Response Phase* revealed significant contralateral gaze-centered directional selectivity of saccades, mainly in the left hemisphere. This is shown in **Figure 9**, which superimposes activity clusters on an inflated brain in the upper row, and their task-specific β weights in the lower row. This includes several additional areas in the parieto-frontal network that did not show gaze-centered directional specificity of target location during the *Delay phase.* In particular, we found higher activation for saccade toward right vs. left in left SEF for both *Control* [*t*_(11)_ = 3.24, *p* = 0.010] and *Landmark Saccade* tasks [*t*_(11)_ = 3.05, *p* = 0.014], middle frontal gyrus (MFG) [*t*_(11)_ = 4.52, *p* = 0.0014], FEF [*t*_(11)_ = 3.59, *p* = 0.0059] and posterior IPS (*p*IPS) [*t*_(11)_ = 4.00, *p* = 0.0031] for the *Control Saccade* task. In addition, there was higher activation for saccade toward right vs. left for the *Landmark Saccade* task in superior parietal lobule (SPL) [*t*_(11)_ = 4.19, *p* = 0.0023], middle temporal gyrus (MTG) [*t*_(11)_ = 3.74, *p* = 0.0046], lateral occipitotemporal gyrus (LOtG) [*t*_(11)_ = 4.13, *p* = 0.0026], anterior (*a*IOG) [*t*_(11)_ = 4.05, *p* = 0.0029] and posterior IOG (*p*IOG) [*t*_(11)_ = 4.18, *p* = 0.0024] in the left hemisphere. Insula in the right hemisphere [*t*_(11)_ = 3.85, *p* = 0.0039] showed higher activation for saccades to left vs. right in the *Landmark Saccade* task. The Talairach coordinates of brain areas were reported in **Table 5**.

**Table 5:** Talairach coordinates and number of voxels for contrast no. 5.

	Talairach coordinates			
		
Brain areas	*x*	*y*	*z*	No. of voxels
**Saccade Right of Gaze > Saccade Left of Gaze**			
Both tasks:				
LH SEF	-5	6	62	440
CS task:				
LH FEF	-24	-6	58	396
LH MFG	-40	9	47	319
LH *p*IPS	-30	-65	32	343
LS task:				
LH SPL	-24	-65	54	394
LH LOtG	-33	-69	-11	392
LH *a*IOG	-47	-60	-1	498
LH *p*IOG	-47	-75	-2	441
**Saccade Left of Gaze > Saccade Right of Gaze**			
LS task:				
eRH Insula	31	25	8	462


In summary, during the *Response phase*, contralateral gaze-centered saccade directional selectivity emerged in occipital, parietal, temporal, and frontal cortex, primarily in the left hemisphere (with the exception of insular cortex).

## Discussion

In this study, we utilized an event-related fMRI design to discriminate between gaze-centered (landmark-independent) vs. landmark-centered (gaze-independent) coding of remembered saccade targets during a *Delay phase* that was temporally and spatially separated (by a pro/anti saccade instruction or a re-presented landmark) from saccade planning and execution (during the *Response phase*). This design differed from saccade tasks where saccade direction was instructed from the beginning of the task. Thus our analysis could focus on the *Delay phase* to distinguish between cortical areas (1) that were differentially activated for gaze-centered vs. landmark-centered target coding, or (2) that showed directional selectivity in either gaze-centered or landmark-centered coordinates.

We used an experimental design where the visual stimuli (including the central fixation point, saccade target and landmark) were present for all the three tasks, the only difference among the tasks was the instruction. Therefore, the purely visual responses should cancel in the comparisons and would not have effect on the observed differences of cortical activation. In the experimental setup, the central fixation point was always displayed at midline and aligned with initial gaze/head/body position in egocentric reference frames. The instruction for participant was to make saccades toward the original target location (a fixed vector relative to the fixation point and initial gaze) in the *Control Saccade* task so that the fixation point would not act as an additional cue. In contrast, participants were instructed to remembered target location relative to a visual landmark and later compute the final saccade direction based on the shifted landmark position in the *Landmark Saccade* tasks. Thus, cortical activity in these two types of saccade tasks would reflect coordinate-dependent saccade target coding.

Our results showed different cortical activity for directional coding of remembered saccade targets, i.e., occipital areas for gaze-centered directional selectivity vs. parietal areas for landmark-centered directional selectivity during the *Delay phase.* Unlike the previous reach study (Chen et al., 2014) showing involvement of temporal and occipital regions in landmark-centered directional selectivity, we observed that spatial specificity in parietal cortex for saccade target coding in the current study. Gaze-centered saccade direction selectivity only appeared in parieto-frontal cortex during the *Response phase*, after movement direction was specified.

### Explicit vs. Implicit Use of Allocentric Cues

Consistent with previous studies (Krigolson and Heath, 2004; Obhi and Goodale, 2005; Krigolson et al., 2007; Chen et al., 2014), here we showed that humans were able to explicitly aim movements toward a location defined relative to a specific allocentric landmark. In other situations, allocentric background information was used implicitly (Whitney et al., 2003; Chen et al., 2011; Uchimura and Kitazawa, 2013), although in these cases motor behavior seemed to only partially weighted toward the allocentric landmark (Byrne and Crawford, 2010). In particular, this weighting seemed to depend on the proximity, number, and perhaps size of background objects (Diedrichsen et al., 2004; Krigolson et al., 2007; Uchimura and Kitazawa, 2013; Fiehler et al., 2014).

Since our design included explicit instructions for spatial coding of targets in the two different tasks (*Control Saccade* and *Landmark Saccade*), it would allow us to contrast areas involved in explicit spatial coding vs. areas involved in implicit spatial coding (Uchimura et al., 2015). In a recent fMRI study that used a non-spatial shape judgment task (that most closely resembles our *Color* control task as opposed to our saccade tasks), Uchimura et al. (2015) found adaptation effects for allocentric stimulus location in precuneus and MOG. These modulations disappeared when the allocentric landmark was reduced to a size comparable to the landmark that was used in the current study. It is difficult to directly compare this study with ours because of task differences (perceptual judgment vs. saccade response), but there are some common elements. In the current study, MOG showed higher activation in the *Color Report* and *Landmark Saccade* tasks than in the *Control Saccade* task, but did not show landmark-centered directional selectivity. Several other areas (including precuneus) did show landmark-centered directional selectivity in the explicit *Landmark Saccade* task, none of these showed implicit landmark-centered directional selectivity in the *Color Report* task. Comparing the results of these two studies suggests that similar (or overlapping) cortical networks are partially activated during implicit allocentric coding (to a degree depending on the salience of the landmark), and fully activated (independent of landmark salience) in tasks that require explicitly allocentric coding. This could explain why landmark proximity, number, and size have different influences on allocentric coding, depending on the task.

### Landmark-Centered vs. Gaze-Centered Cortical Activation during the *Delay Phase*

In most previous fMRI studies of egocentric coding for saccades, movement direction was instructed from the beginning of each trial (Connolly et al., 2002; Medendorp et al., 2003, 2006; Schluppeck et al., 2005; Van Pelt et al., 2010). As noted above, our study differed in that participants did not know which way they would saccade until the *Response phase*, enabling us to focus our analysis on remembered target coding during the *Delay phase.*

We found overlapping areas in parietal and frontal cortex in the two saccade tasks (*Control Saccade*, *Landmark Saccade)* as opposed to the non-spatial control task (*Color Report)* during the *Delay phase*. Time course data show that preparatory activation for saccades diverged from the visual activity for the *Color Report* task as early as 2 s of the delay phase. Some of these parieto-frontal areas were different from those for reach tasks from our previous reach study (Chen et al., 2014) because participants had been pre-cued about what type tasks to perform before the delay phase, which would influence the general preparatory activity related to the effector (arm vs. eye). We did not do statistical analysis to directly compare these two studies, because the task detail was not identical as well as the recruited participants were not same.

Our results showed that temporal cortex (ITG) and occipital cortex (calcarine, cuneus and MOG) were preferentially involved in landmark-centered saccade target coding. Although these occipital areas also showed higher activation in the *Color Report* as compared to the *Control Saccade* task, ITG only showed higher activation in the *Landmark* vs. *Control* saccades, suggesting temporal cortex was selective for spatial memory of landmark-centered saccade targets in our study. Temporal cortex has previously been implicated in allocentric coding in neuropsychological studies (Milner and Goodale, 2006; Schenk, 2006), whereas occipital cortex (calcarine, cuneus, and MOG) is generally thought to be involved in stimulus-feature processing and visual working memory (Greenlee et al., 2000; Harrison and Tong, 2009). Unlike neuroimaging studies of human navigation, we did not observe higher activation in retrosplenial cortex in our landmark-centered saccade task. The reason could be that retrosplenial cortex is involved in spatial coding of scene in a large-scale familiar environment (Epstein, 2008; Sulpizio et al., 2013; Miller et al., 2014). However, our landmark-centered saccade task differed from spatial navigational tasks where two different types of allocentric reference frames were used for spatial coding, i.e., landmark-centered vs. environment-centered.

In contrast, right *a*mIPS and SMG were preferentially involved in gaze-centered saccade target coding. Area mIPS is thought to correspond to the human parietal eye fields (Muri et al., 1996; Sereno et al., 2001; Medendorp et al., 2003, 2005a; Koyama et al., 2004; Pierrot-Deseilligny et al., 2004; Hagler et al., 2007; Merriam et al., 2007; Vesia et al., 2010) and to correspond to monkey lateral intraparietal cortex (Andersen and Buneo, 2002; Culham et al., 2006; Vesia and Crawford, 2012), whereas SMG is thought to be involved in spatial memory (Moscovitch et al., 1995; Salmon et al., 1996; Faillenot et al., 1997; Silk et al., 2010).

### Directional Selectivity for Saccade Target Coding during the *Delay Phase*

Previous neuroimaging studies indicated that human mIPS and FEF preferentially code contralateral saccade target in egocentric coordinates (Medendorp et al., 2003, 2005b; Curtis and D’Esposito, 2006; Kastner et al., 2007). However, the design of those studies may have conflated saccade target memory and planning. Previous neurophysiological studies have shown that neurons in lateral intraparietal sulcus (LIP) and SEF can code saccade target location within an object relative to other parts of the same object (object-centered coordinates), with a weaker signal in the former (Sabes et al., 2002; Olson, 2003). But to our knowledge, the cortical activity for spatial selectivity of saccade target memory in gaze-centered and landmark-centered (target relative to a separate visual landmark) reference frames have not been studied before the current investigation.

In the present study, during the *Delay phase* a preference for contralateral saccade targets relative to gaze was observed in right SOG and IOG in the *Control Saccade* task (note again that gaze, head, and body coordinates were aligned with midline; we made no attempt to distinguish between these egocentric frames in this experiment). Similar brain areas in occipital cortex for the egocentric directional selectivity of reach target memory were reported in our previous reach study (Chen et al., 2014), which used a similar design except for details of the fixation requirements, timing, and of course the effector used for the final action. We did not do a direct statistical comparison of the data from these two studies because of these minor design differences and because different pools of participants were employed.

In comparison, we found landmark-centered directional selectivity in bilateral precuneus and left mIPS. This specific direction specificity in the landmark-centered coordinates cannot be due to anticipation of re-appearance of the allocentric landmark, because the future location of this cue was unpredictable in our task. The involvement of PPC areas in allocentric directional selectivity of saccade targets is consistent with the suggestion of non-retinal representation of target location for saccades in PPC from neurophysiological (Galletti et al., 1993; Thier and Andersen, 1996; Mullette-Gillman et al., 2005) and human imaging studies (Pertzov et al., 2011). For instance, Pertzov et al. (2011) indicated that the multiple reference frames in mIPS for saccade target coding could be head-centered, body-centered, or even allocentric coordinates. However, that study did not distinguish between non-retinal egocentric and allocentric frames of reference. Previous fMRI study found the involvement of precuneus in both encoding and retrieval of allocentric spatial locations using spatial memory tasks (Frings et al., 2006). Although that study did not use action-related tasks and did not take into account the allocentric directional selectivity, it has suggested the role of precuneus in encoding target locations in allocentric coordinates in memory. Our finding further indicates that precuneus is involved in landmark-centered directional selectivity of remembered saccade targets.

In contrast to our previous study (i.e., rather than observed landmark-centered directional selectivity in inferior occipital and ITG for reach targets), we found precuneus and mIPS showing direction specificity for saccade targets in landmark-centered coordinates. This difference might have something to do with the speed and frequency of saccades relative to relatively sluggish reaches, perhaps requiring a more direct link between allocentric and egocentric coding mechanisms (Crowe et al., 2008). Unlike previous neurophysiological studies showing object-centered saccade target coding in SEF (Olson and Gettner, 1995, 1996; Olson and Tremblay, 2000), we did not observe landmark-centered directional specificity in SEF in our study. This could reflect the difference between the two non-egocentric reference frames used in these studies (independent allocentric landmark vs. intrinsic object-centered) as well as the different techniques (i.e., fMRI in untrained human subjects vs. unit recording in trained monkeys), thus could suggest the divergent neural mechanisms related to each of them for the coding of saccade target direction relative to an external landmark vs. to a part of the object itself.

### Direction Selectivity in *Delay* vs. *Response Phases*

It is important to point out that the spatial details of saccade planning and execution could only occur in our *Response phase* after movement direction was specified by re-appearance of the landmark in the *Landmark Saccade* task and by providing a *pro/anti-saccade* auditory cue in the *Control Saccade* task right before the *Response phase*. This would explain why, unlike previous fMRI studies showing contralateral directional selectivity in parietal and frontal cortex for saccades (Medendorp et al., 2003; Kastner et al., 2007), we did not observe any gaze-centered directional selectivity in the parieto-frontal network during our *Delay phase*. As noted before, except for analyzing saccade directional selectivity in the *Control Saccade* task, we also did it in the *Landmark Saccade* task to test whether different brain regions would be activated when a shifted landmark was used for re-computation of the saccade vector.

As expected, we found directional selectivity contralateral to the direction of saccades in several parietal and frontal areas in the left hemisphere, such as SEF in both *Control* and *Landmark Saccade* tasks, FEF and *p*IPS in the *Control Saccade* task and SPL in the *Landmark Saccade* task. The observed saccade directional selectivity in left SEF for both *Control* and *Landmark Saccade* tasks suggests that SEF could play a role in transforming gaze-centered and landmark-centered coding into saccade commands. We were somewhat surprised by the additional recruitment of left occipital and temporal areas for directional selectivity of rightward saccades in the *Landmark Saccade* task. These areas are not normally associated with control of saccades. This might reflect the greater degree of task complexity, and/or the maintenance of allocentric coding mechanisms during the *Response phase*. Likewise, we were somewhat surprised to find that only the right insula showed directional selectivity of leftward saccades in the *Landmark Saccade* task. This directionally selective activation might be related to a role of right insula in more complex saccade tasks (Blurton et al., 2012), like the *Landmark Saccade* task in our study.

We observed a similar pattern of contralateral directional selectivity for saccades to that for reaches during the *Response phase*, with more areas in the left hemisphere (Chen et al., 2014). This is easier to explain for reaches as interactions between visual directional selectivity and contralateral hand specificity (Perenin and Vighetto, 1988; Rossetti et al., 2003; Medendorp et al., 2005a; Beurze et al., 2007; Blangero et al., 2007; Vesia and Crawford, 2012), but hemispheric specialization for saccadic eye movements is still debated (Pierrot-Deseilligny et al., 1991; Muri et al., 2000; Leff et al., 2001; Muri et al., 2002; Yang and Kapoula, 2004). However, it has been suggested that saccade-related hemispheric asymmetry in PPC could be influenced by factors such as latency and dynamics (Yang and Kapoula, 2004; Vergilino-Perez et al., 2012). One thing conspicuously missing in both our *Delay* and *Response* data was contralateral selectivity in the superior colliculus (Wurtz and Albano, 1980; Munoz, 2002; Gandhi and Katnani, 2011; Sadeh et al., 2015), but this is likely related to the limitations of standard fMRI techniques in revealing subcortical activation.

### Methodological Limitations and Potential Neural Mechanisms

Functional MRI has been a popular technique used in examining sensorimotor functions because of its relatively high spatial resolution and capacity to explore the entire network of brain regions for particular tasks (Alkan et al., 2011; Thaler and Goodale, 2011; Leoné et al., 2014, 2015; Miller et al., 2014; Fiehler et al., 2015; Fabbri et al., 2016; Hutchison and Gallivan, 2016; Straube et al., 2017). However, fMRI also has its limitations that have to be considered when interpret data. First, functional activation of the brain is reflected by hemodynamic response, the blood-oxygen-level dependent (BOLD) signal, which cannot directly measure action potentials in single neurons, and may in fact be more closely related to sub-threshold, post-synaptic multiunit activity (Goense and Logothetis, 2008; Lippert et al., 2010). Second, the time course of the BOLD response is too sluggish to reflect the exact timing of neural events. However, with these caveats in mind, one can speculate about what one might find if one recorded from the relevant neurons in this task.

First, the general tendency for contralateral directional selectivity in the gaze-centered saccade task can be explained by the propensity, if not always exclusivity, of contralaterally organized visual and motor response fields in task-related neurons extending from occipital to frontal cortex (Crowne et al., 1981; Mishkin and Ungerleider, 1982; Bruce and Goldberg, 1985; Andersen et al., 1990; Blatt et al., 1990; Sajad et al., 2015, 2016). The more difficult question is how to implement the landmark-centered coding schemes observed here. It may be that neurons encode the target and landmark separate, but another possibility is that visual receptive fields become anchored on the landmark, but are extended toward the target, similar to object-centered results in the SEFs (Olson and Tremblay, 2000; Olson, 2003) and extension of receptive fields during tool use (Iriki et al., 1996; Maravita and Iriki, 2004). Yet another possibility is that target position responses are modulated by landmark ‘gain fields’ or vice versa (Andersen and Mountcastle, 1983; Zipser and Andersen, 1988). Finally, this could be implemented by the type of single unit and population responses that have been observed in the hippocampus, but have not been reported in the saccade system (Eichenbaum et al., 1989; Shapiro et al., 1997). Importantly though, our results suggest that this does not simply happen within the cortical sites activated by standard saccade tasks, but rather involves activity with and between other sites. This suggests some ‘binding mechanism’ is involved, specifically to bind information between the landmark and the target. A potential mechanism for this is synchronization of subthreshold and/or suprathreshold neuronal activity between sites (Engel et al., 1991; Lewis et al., 2016). All of this must remain speculative until neurophysiological recordings are performed, but at least the current results point toward regions where such recordings might yield results that are relevant for this question, and conversely, where damage might cause deficits that are clinically observable.

In summary, other than the few exceptions noted above, the cortical activation observed during the *Response phase* was generally consistent with previous fMRI literature on egocentric movement selectivity for saccades and the ways this differs from reach direction selectivity (Beurze et al., 2007; Fernandez-Ruiz et al., 2007; Busan et al., 2009; Chen et al., 2014). This difference is in accordance with effector specificity for reach vs. saccade planning and execution (Medendorp et al., 2005a; Connolly et al., 2007; Beurze et al., 2009; Vesia et al., 2010). Likewise, as noted above we found some detailed differences between directional selectivity for saccade and reach target memory in our current and previous studies (Chen et al., 2014). But the important common message from both our studies is that cortical activation for target coding at gaze-centered and landmark-centered coordinates differs, both from each other and from the cortical mechanisms used for the planning and execution of movement.

## Author Contributions

YC, designed the experiment, recruited participants, collected, analyzed and interpreted data, wrote the paper, agreed to be accountable for all aspects of the work. JC, designed the experiment, analyzed and interpreted data, wrote the paper, agreed to be accountable for all aspects of the work.

## Conflict of Interest Statement

The authors declare that the research was conducted in the absence of any commercial or financial relationships that could be construed as a potential conflict of interest.
